# Optimizing Water Volume for Carrots (*Daucus carota*) Grown in a Deep-Water Culture System

**DOI:** 10.3390/plants15071101

**Published:** 2026-04-03

**Authors:** Dario Rueda Kunz, Haydee Laza, Jyotsna Sharma, Marcos X. Sanchez-Plata, Catherine Simpson

**Affiliations:** 1 Plant and Soil Science Department, Texas Tech University, 2911 15 Street, Lubbock, TX 79049, USA; daruedak@ttu.edu (D.R.K.); haydee.laza@ttu.edu (H.L.); jyotsna.sharma@ttu.edu (J.S.); 2 Animal and Food Science Department, Texas Tech University, 1308 Indiana Ave, Lubbock, TX 79415, USA; marcos.x.sanchez@ttu.edu

**Keywords:** hydroponics, carrot, nutrient solution volume, water use efficiency, taproot development

## Abstract

Efficient water management is essential for sustainable agriculture, particularly for crops like carrots that are traditionally grown in soil systems with high water consumption, averaging 4500–6000 m^3^/ha. Hydroponics offers a potential alternative due to its higher water-use efficiency, yet root crops have been understudied because of system design and economic challenges. This study evaluated the effects of different hydroponic solution volumes on the growth of carrots (*Daucus carota* cv. Mokum) in a Deep-Water Culture (DWC) system to address knowledge gaps regarding their feasibility in soilless production. Experiments were conducted in a controlled greenhouse using three solution volume treatments (50% with 10.75 L, 75% with 16.13 L, and 100% with 21.50 L) applied to 12 plants per treatment across two repeated experiments. Biomass production, water use efficiency, and total carotenoid concentration were assessed after eight weeks. The 100% (21.50 L) volume treatment produced the greatest shoot and root biomass, whereas the 50% (10.75 L) volume treatment significantly increased total carotenoid concentration, particularly in the second trial. Despite lower water inputs, water use efficiency did not differ statistically among treatments. These results indicate that carrots can be successfully cultivated in DWC systems, though further optimization, such as using narrower containers, may be required to improve efficiency and competitiveness with soil-based production.

## 1. Introduction

Water is a vital resource for agriculture [1], making it essential to utilize natural resources effectively for crop growth [2]. Approximately 71% of the Earth’s surface is covered by water, with 96.5% of that found in the oceans [3]. The remaining freshwater is distributed among sources like polar ice caps, glaciers, permanent snow, groundwater, lakes, rivers, streams, soil, and water vapor [4]. This freshwater is estimated to total around 10 million cubic kilometers, yet less than 32% is easily accessible for human consumption and agricultural use [4]. However, traditional soil-based farming methods often struggle with water efficiency [5]. This inefficiency originates from various factors, including excessive evaporation from the soil, poor soil structure, climate conditions, and outdated irrigation practices [6].

Hydroponic production can increase water use efficiency and conserve water resources [7]. Some of these systems have been reported to reduce water use by up to 114% compared to open-field production, but water savings are highly dependent upon crop and system [8]. Hydroponic systems include flood and drain, trickle feed, nutrient film technique (NFT), aeroponics, and deep-water culture (DWC) [9]. While different hydroponic systems exhibit varying levels of efficiency, a study by Majid et al. [10] found that NFT was significantly more efficient than DWC for lettuce (*Lactuca sativa*) production. Additionally, the efficiency of DWC was closer to that of soil-based systems than that of NFT. The significant difference is likely attributed to the practices associated with DWC. In this method, frequent water changes and having fewer plants per area can reduce efficiency. Additionally, lettuce has a shallow root system, which, having excessive tub sizes for DWC, could reduce the efficiency of the plant roots accessing the water and nutrients [10].

One possible solution to improve the efficiency of DWC is to reduce the water used during crop growth. Additionally, keeping the taproot from being completely submerged in the nutrient solution may enhance root enlargement and lead to higher yields. This has been demonstrated in other root crops using hydroponic methods [11,12,13,14]. For instance, studies on sweet potatoes (*Ipomoea batatas*) have shown that using sandy medium-based systems [11,14], rockwool slab-based systems [12], and two-layer systems with vermiculite and nutrient solution [13] resulted in an increase in root growth when no nutrient solution surrounded the roots.

While some research has investigated taproot growth in carrots (*Daucus carota*) within soilless culture systems [15,16,17,18], none has specifically examined the effects of reducing the volume of nutrient solution in the reservoir. Understanding the findings from previous soilless culture studies is, therefore, essential for contextualizing this gap. Gichuhi and collaborators [15] reported the effects on carrot growth when grown on an NFT and a microporous tube membrane system (MTMS). The MTMS is a soilless culture technique in which the crop grows in a substrate, such as turface, and nutrient solution is circulated through a porous tube, with the roots taking up water and nutrients via capillary movement, as reported first in 1998 using sweet potato [19]. In comparing NFT and MTMS, the authors reported greater growth in the MTMS experiments, as evidenced by the visual appearance of coiled, branched taproots in the NFT system [15]. Another soilless experiment using rockwool, with and without holes, during carrot growth revealed that the presence of holes filled with vermiculite in the rockwool aided the formation and growth of the taproot [16]. Whereas the effect of aeration time and whole roots or only lateral root submersion showed that increasing the aeration period of the nutrient solution improved the tap root enlargement, additionally, when only the lateral roots were submerged in the constantly aerated nutrient solution, an increase in weight and enlargement was reported [18]. Lastly, a more recent study in which carrot plants were grown with and without aeration in a hydroponic system demonstrated that the absence of aeration of the nutrient solution was detrimental to taproot formation and enlargement. Additionally, hypoxia led to increased lignin levels [17].

Carrot production worldwide has nearly doubled since 2003, increasing from 25 million to 41 million tonnes [20]. In contrast, production in the United States has decreased by 20% over the same 20 years, falling to 1.3 million tonnes [20]. Despite this decline in production volume, the value of carrot production in the U.S. has risen by 260% [21]. This indicates that the growth of carrots in the U.S. is significant in terms of monetary value, likely due to their nutritional benefits [22]. Carrots are rich in carotenoids and anthocyanins, contributing to their distinctive orange, yellow, red, and purple tap root colors [23]. In addition, they also contain a unique combination of three flavonoids: kaempferol, quercetin, and luteolin [24,25,26]. Other beneficial compounds, such as vitamin E and ascorbic acid [27], can also be found in carrots, and according to 2010 data, the average total carotenoid (TC) content in U.S. carrots was reported as 135 ppm [28].

To address these gaps, this study evaluated carrot growth in a deep-water culture hydroponic system using different reservoir water volumes to study impacts on water use efficiency, yields, and carotenoid concentrations in carrots cultivated within this system.

## 2. Results

**Figure 1:**
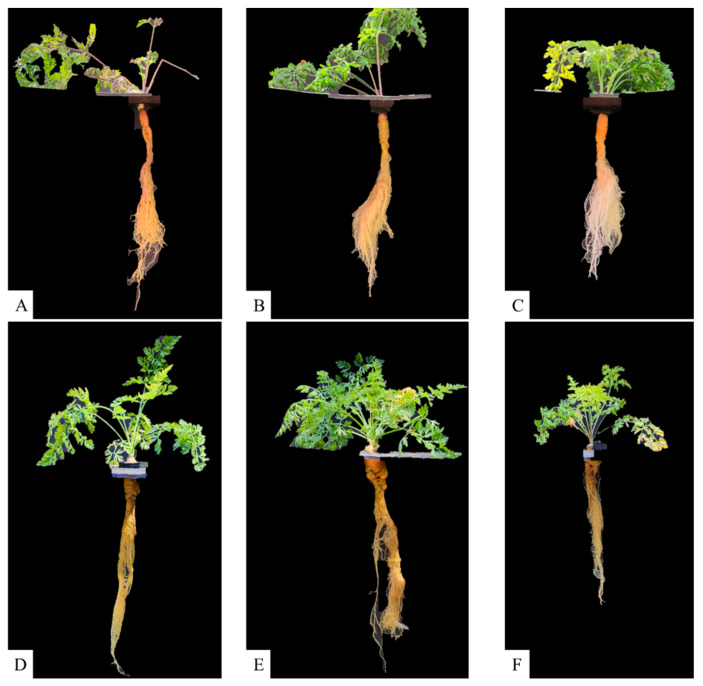
Carrot plants grown in a hydroponic deep-water culture system with different nutrient solution volumes. Experiment 1: (**A**) 50% (10.75 L) volume, (**B**) 75% (16.13 L) volume, (**C**) 100% (21.50 L) volume. Experiment 2 (**D**) 50% (10.75 L), (**E**) 75% (16.13 L) volume, (**F**) 100% (21.50 L) volume. Plants show represent the average taproot weight.

To evaluate the effects of reduced nutrient solution volume on carrot deep-water culture, above- and below-ground biomass production and total carotenoid concentration were measured under controlled environmental conditions. As illustrated in Table 1 and Figure 1, using 100% (21.50 L) volume treatment on both experiments resulted in the greatest aerial biomass weight, with no significant difference between treatments. Additionally, aerial height showed no significant difference between treatments in either experiment. Although the 50% (10.75 L) volume treatment had a reduction of 33% in tap root weight and 45% in lateral root weight at harvest when compared with the 75 % (16.13 L) volume treatment and 100 % (20.50 L) volume treatment, respectively, the decline was not significant in experiment 1 (Table 1 and Figure 1). A similar situation was documented in experiment 2 for the 50% (10.75 L) volume treatment, where reductions of 22% in tap root weight and 23% in lateral root weight were observed at harvest compared with the 75% (16.13 L) and 100% (20.50 L) volume treatments, respectively (Table 1 and Figure 1).

If we take the total root biomass (taproot weight + lateral root weight) and divide it by the area of production for each treatment in experiment 1, for the 50% (10.75 L) volume treatment it obtained a yield of 0.150 kg/m^2^, for the 75% (16.13 L) volume treatment it obtained 0.238 kg/m^2^, and for the 100% (20.50 L) volume treatment it obtained 0.250 kg/m^2^. Now, for experiment 2, the 50% (10.75 L) volume treatment yielded 0.213 kg/m^2^, the 75% (16.13 L) volume treatment yielded 0.210 kg/m^2^, and the 100% (20.50 L) volume treatment yielded 0.274 kg/m^2^.

For total carotenoids, the 50% (10.75 L) volume treatment resulted in a 20% and 60% increase in total carotenoids for experiments 1 and 2, respectively, compared to the 100% (21.50 L) volume. Only experiment 2 showed a significant increase with *p* ≤ 0.01 (Table 1 and Figure 1). Nevertheless, the efficiency of water use, measured as fresh tap root weight per total liters of water consumed, remains statistically the same among the treatments due to the compensation effect. The 50% (10.75 L) volume treatment had significantly lower water consumption than the 75% (16.13 L) and 100% (21.50 L) volume treatments in experiment 1 (*p* ≤ 0.1), but no significant differences were found in experiment 2. Lower production resulted in lower water consumption; conversely, higher production led to higher consumption in both experiments (Table 2).

WinRhizo analysis was performed on the second experiment for tap and lateral roots, including total length, average diameter, and root volume. The 75% (16.13 L) volume treatment had a significantly higher average diameter at *p* ≤ 0.05 and a higher root volume, although this latter variable was not significant at *p* ≤ 0.05 (Table 3).

**Figure 2:**
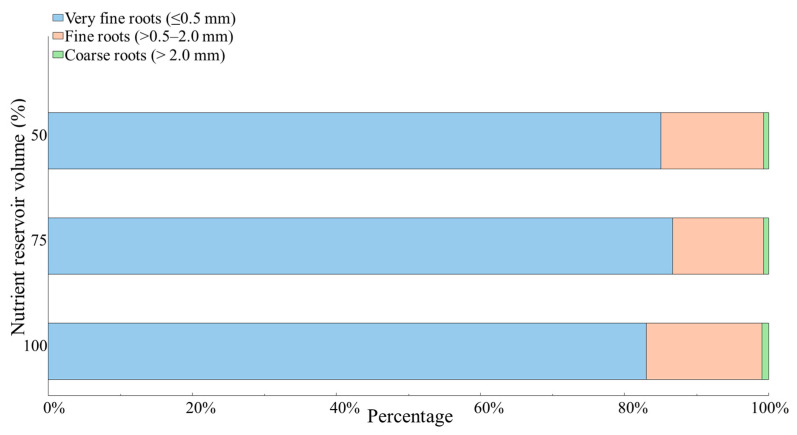
Root classification distribution by nutrient volume reservoir treatment of carrots grown in a deep-water culture system.

Figure 2 shows the distribution of root thickness categories—very fine, fine, and coarse roots—during experiment 2 for each volume treatment. Very fine roots made up at least 83% of the carrot roots across all three treatments, with the highest concentration in the 75% (16.13 L) volume treatment. In contrast, coarse roots (greater than 2.0 mm) were present in the lowest quantities among the treatments, with the highest percentage recorded at just 0.92% for the 100% (21.50 L) volume treatment. Interestingly, the same treatment also had the highest rate of fine roots (ranging from 0.5 to 2.0 mm), accounting for 16.07%.

Interestingly, when a multivariate correlation analysis is conducted, water use efficiency and TC exhibit a weak, negative linear correlation in experiments 1 and 2, although this correlation is not significant at *p* < 0.05 (Table 4 and Table 5). Moreover, TC was only negatively and significantly correlated with aerial weight, lateral root weight, and the lateral root/taproot ratio, but only in experiment 1. Conversely, and as expected, a high positive significance correlation was observed between aerial weight and lateral and tap root weights (Table 4 and Table 5). Lastly, total water consumption showed no significant correlation with any measured variable in both experiments, besides the root/shoot ratio in experiment 1.

## 3. Discussion

This study evaluated the viability of growing carrots in a DWC hydroponic system by testing three different nutrient solution volumes and assessing their impact on carrot growth, water use efficiency, and total carotenoid content. While DWC systems are commonly used for leafy greens [29], their application to root crops like carrots remains underexplored. These results confirmed that DWC can support carrot cultivation, with a 97% survival rate and tap root formation for harvest. Although the average taproot weight (15–50 g) was lower than that reported for soil-based systems [30,31], it was comparable to or exceeded the weights (4–26 g) observed in other hydroponic studies [32,33]. This suggests that further optimization is required to match or exceed traditional yields.

These results indicate that the volume of the nutrient solution significantly affects root development. Carrots grown in full-volume reservoirs produced more uniform and heavier taproots, while reduced volumes led to uneven root development. This was likely due to increased root exposure to air, which limited water availability and hindered lateral root formation. Since primary root growth is indeterminate and secondary and tertiary roots are determinate, insufficient water can halt growth and cause tissue death [34]. Interestingly, reduced nutrient volumes were associated with increased total carotenoid concentrations in the taproots, particularly in experiment 2, where the 50% (10.75 L) volume treatment resulted in a 60% increase in total carotenoids. This suggests that mild water stress may stimulate carotenoid biosynthesis, possibly as a response to oxidative stress. Similar findings have been reported in drought-stressed carrots [35] and other crops, such as lettuce, where stress responses influenced yield and nutrient content [36]. The elevated carotenoid levels may also be linked to increased reactive oxygen species in roots exposed to air [37].

In terms of resource efficiency, water-use analysis revealed that while plants in lower-volume treatments consumed less water, their water-use efficiency remained statistically unchanged. This was likely due to a compensatory effect where lower biomass production corresponded with lower water consumption, and vice versa. The initial hypothesis was lower water volumes in the DWC reservoir would result in a higher water use efficiency, but reducing the water volume did not improve water use efficiency, and the DWC system was less efficient than traditional soil-based methods [38] and soilless production methods other than hydroponics [39]. For instance, even when hydroponics is a soilless production system, the calculated efficiency levels (0.52–2.7 g/L) are between 7 and 57 times smaller than previously reported in the literature (20–30 g/L), where substrate was used for the growth of carrots [39], suggesting that the absence of the substrate in this study may have impacted the efficiency. In soil-tested water-use efficiency for carrots, the range has been reported as 15–47 kg/m^3^, which is 5 to 90 times higher than in this hydroponic carrot study [38]. Moreover, this contrasts with findings in other hydroponic crops, such as tomatoes [8], lettuce [10], and watercress [40], where hydroponic systems often outperformed soil-based ones. This behavior in other crops could be associated with the harvestable tissue obtained, which can be higher in volume and mass. Additionally, the edible product is not located underground, and in a system like hydroponics, successful growth and enlargement of tubers, roots, and other tissues can present challenges. Finally, a direct comparison with other hydroponic studies on water-use efficiency in carrots is not possible due to the limited information available in the literature.

From a practical standpoint, optimizing the volume of the nutrient solution is crucial for balancing yield, water use, and nutritional quality in hydroponic carrot production. While higher water volumes (75%: 16.13 L; 100%: 21.50 L) promote biomass, lower volumes (50%: 10.75 L) may enhance nutritional value through increased carotenoid content. However, water volume is often a function of the container. Because carrots have long, slender roots, a nutrient reservoir or container that has similar characteristics may be a better way to reduce volume, but this would also restrict the number of plants per container. These trade-offs must be carefully considered in commercial applications. Future research should investigate additional variables, such as aeration rates, nutrient formulations, and economic feasibility, to further refine DWC systems for root crops. Integrating physiological, agronomic, and economic data will be essential to developing sustainable and scalable hydroponic solutions for carrot production.

## 4. Materials and Methods

### 4.1. Experimental Design

**Figure 3:**
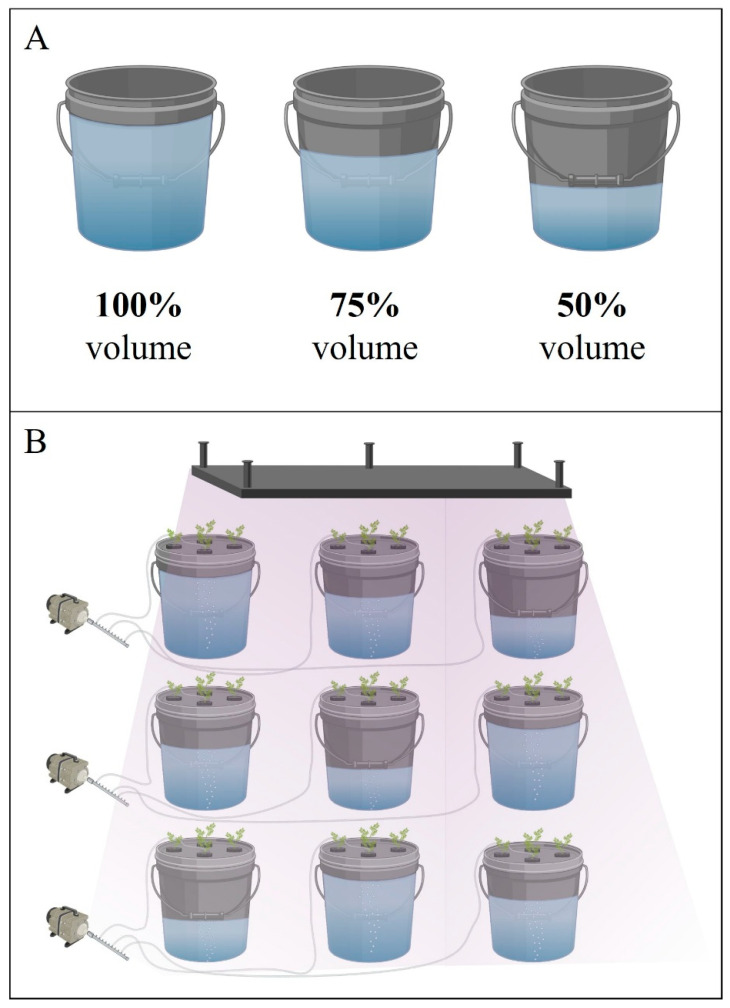
(**A**) Visual representation of the treatments applied to the buckets during deep-water culture. (**B**) Experimental design layout for both experiments. Figure created using BioRender.com. Accessed on 30 March 2026.

The experiment was conducted in two separate experiments in a controlled greenhouse environment at the Texas Tech University Horticulture Gardens and Greenhouse Complex. Carrot (*Daucus carota*) seeds were germinated in a vermiculite and perlite (1:1) substrate mixture and grown until the seedlings were at the second true leaf stage. In addition to watering every 2 days, 50 ppm fertilization with OASIS^®^ Hydroponic Fertilizer 16-4-17 (Kent, OH, USA) was conducted once before transferring the seedlings into the DWC hydroponic system. Carrot plants were grown for 68 days after transfer (DAT) for experiment 1 (July 2023) and 62 DAT for experiment 2 (March 2024) in the DWC hydroponic system. The DWC system was set up using five-gallon (18.92 L) buckets, with nine buckets allocated for each experiment, allowing four plants to be placed in each bucket. Carrot plants were suspended in the bucket using foam collars. Air bubbles were supplied to the nutrient solution via an air pump to ensure proper aeration. The nutrient solution mix, prepared using OASIS^®^ fertilizer, maintained a concentration of 200 ppm N. To promote optimal plant growth, the water in the buckets was replaced every two weeks. The treatments utilized three distinct water volumes in the buckets: 50% (10.75 L), 75% (16.13 L), and 100% (20.50 L). When the seedlings were transferred, all buckets received a full-volume treatment of 100% (21.50 L) to help them acclimate and allow young roots to reach the nutrient solution. After four weeks, three buckets were maintained at 100% (21.50 L) volume, while the next three buckets were adjusted to 75% (16.13 L) volume, and the final three were reduced to 50% (10.75 L) volume treatment (Figure 3A). Thus, each treatment consisted of a total of 12 plants, resulting in 36 plants in total for each experiment. The experimental design layout was composed of a Latin Square design (Figure 3B).

Supplemental lighting was provided using the LumiGrow TopLight Node™ (Emeryville, CA, USA), with a minimum Daily Light Integral (DLI) target of 20 mol/m^2^/d. Light intensity was measured using a PAR meter and recorded with a HOBO datalogger (Bourne, MA, USA), which also tracked temperature and humidity levels. During the plant growth phase, the average greenhouse temperature was 27.4 °C for experiment 1 and 25.7 °C for experiment 2. In experiment 2, additional temperature sensors were implemented to monitor water temperatures throughout the experiment.

### 4.2. Measurements

At harvest, indicators such as aerial weight, plant height, tap and lateral root weight, and whole root length were measured. The tap roots were then sent to the lab for freeze-drying and analysis of total carotenoids. For experiment 2, an additional WinRhizo analysis was also conducted on tap and lateral roots before freeze-drying. Finally, the freeze-dried carrot taproots were ground into a fine powder for analysis of total carotenoid concentration.

WinRhizo analysis was performed on tap and lateral roots, which were kept in a 1:1 ratio of ethanol and DI water overnight to prevent decay. The carrot roots were scanned using the Epson Perfection V850 Pro Photo Scanner and analyzed using WinRhizo Pro v. 2016a software. Variables obtained included total length, average diameter, root volume, and root length, categorized by diameter groups into very fine, fine, and coarse roots.

### 4.3. Chemical Analysis

The total carotenoid analysis was conducted as previously described [41], with minor adjustments. In brief, 50 mg of ground carrot powder was measured into 5 mL tubes, to which 3 mL of HPLC-grade methanol was added and mixed thoroughly by vortexing. After ensuring the tissue and solvent were thoroughly combined, the mixture was sonicated for 15 min using an ultrasonic homogenizer. Then, the samples were centrifuged for 10 min at 5000 rpm, with the centrifuge pre-cooled to 4 °C. Finally, the absorbances of 200 μL of supernatant were measured using the SpectraMax iD3 spectrophotometer (Molecular Devices LLC, San Jose, CA, USA) at wavelengths of 750, 665, 652, and 470 nm. The first wavelength confirmed a clear supernatant, while the next three absorbances were analyzed using the designated equations below [41].

(1) $$c_{a}\;(\mu g/\mathrm{mL})=16.72\;A_{665}-9.16\;A_{652}$$

(2) $$c_{b}\;(\mu g/\mathrm{mL})=34.09\;A_{652}-15.28\;A_{665}$$

(3) $$c_{\left(x+c\right)}\;(\mu g/\mathrm{mL})=(1000\;A_{470}-1.63\;c_{a}-104.96\;c_{b})/221$$

After obtaining the concentration from Equation (3), it was multiplied by 60 to calculate the total carotenoid concentration in μg/g dry weight (DW).

### 4.4. Statistical Analysis

The two experiments were conducted in two different seasons, therefore data from each experiment were analyzed independently using analysis of variance (ANOVA) to assess the effects of nutrient solution volume (50%: 10.75 L, 75%: 16.13 L, and 100%: 21.50 L) on plant performance metrics, including aerial biomass, root biomass, aerial height, maximum root length, and total carotenoid content in taproots (For statistical calculations in both experiments we had at least n = 11 for each treatment). To account for potential correlations among response variables, residual maximum likelihood (REML) and Pairwise methods were used to examine multivariate relationships where appropriate. All analyses were performed using JMP Pro version 18 (SAS Institute Inc., Cary, NC, USA), and a significance level of α = 0.05 was used to determine statistical differences.

## 5. Conclusions

This research demonstrates the feasibility of growing carrots in a DWC hydroponic system, with 97% plant survival and taproot weights averaging 19 and 43 g at harvests. The study revealed that using the full capacity of the nutrient solution reservoir is more conducive to carrot root growth. At the same time, reduced volumes by 50% (10.75 L) can enhance carotenoid concentration due to potential water stress. However, the overall water use efficiency of the DWC system did not exceed that of traditional soil-based systems. These results underscore the need for further refinement of hydroponic methods for root crops to achieve sustainability and efficiency comparable to those of conventional agriculture. Future research should focus on optimizing nutrient solution management and exploring the physiological responses of carrots to hydroponic conditions to improve yield and quality.

## Figures and Tables

**Table 1:** Effects of the nutrient solution volume on carrot growth with a deep-water culture system.

Treatment	Aerial Weight	Taproot Weight	Lateral Root Weight	Aerial Height	Max Root Length	Total Carotenoids	Root/Shoot Ratio	Lateral Root/Tap Root Ratio
	(g)	(g)	(g)	(cm)	(cm)	(μg/g DW)		
First experiment								
50% (10.75 L) Volume	16.02	15.03	17.86	20.04	40.56	134.09	2.13	1.55
75% (16.13 L) Volume	17.93	22.56	29.57	18.55	49.71	125.84	2.63	0.96
100% (21.50 L) Volume	20.80	21.91	32.74	19.21	44.76	111.44	2.74	1.39
*p*-values	0.494 ^NS^	0.110 ^NS^	0.156 ^NS^	0.724 ^NS^	0.253 ^NS^	0.550 ^NS^	0.075 ^NS^	0.204 ^NS^
Second experiment								
50% (10.75 L) Volume	8.63	39.61	7.09	12.52	48.58 ^a^	266.54 ^a^	5.68	0.18
75% (16.13 L) Volume	6.95	39.52	6.34	11.79	39.08 ^b^	218.47 ^ab^	6.21	0.14
100% (21.50 L) Volume	9.84	50.65	9.26	12.58	34.37 ^b^	166.08 ^b^	5.76	0.16
*p*-values	0.174 ^NS^	0.205 ^NS^	0.200 ^NS^	0.548 ^NS^	0.007 **	0.005 **	0.511 ^NS^	0.219 ^NS^

^i^ Mean separation is shown by different lowercase letters in columns as indicated by Tukey HSD test at *p* ≤ 0.05. ^ii NS^ indicate that the F values for 50% (10.75 L), 75% (16.13 L), and 100% (21.50 L) volume treatments are not significant (*p* > 0.05); ** indicate that the F values for 50% (10.75 L), 75% (16.13 L), and 100% (21.50 L) volume treatments are significant at *p* ≤ 0.01.

**Table 2:** Water consumption and WUE during carrot growth with a deep-water culture system.

Treatment	Water Consumption Before TRT	Water Consumption During TRT	Total Water Consumption	Water Use Efficiency
	(L)	(L)	(L)	(g FW/L)
First experiment				
50% (10.75 L) Volume	15.18	13.62 ^b^	28.80	0.52
75% (16.13 L) Volume	15.36	16.43 ^ab^	31.79	0.72
100% (21.50 L) Volume	14.86	20.00 ^a^	34.86	0.63
*p*-values	0.822 ^NS^	0.039 *	0.124 ^NS^	0.293 ^NS^
Second experiment				
50% (10.75 L) Volume	8.93	7.29	16.22	2.44
75% (16.13 L) Volume	8.89	7.32	16.21	2.44
100% (21.50 L) Volume	9.21	9.49	18.69	2.70
*p*-values	0.796 ^NS^	0.066 ^NS^	0.143 ^NS^	0.74 ^NS^

^i^ Mean separation is shown by different lowercase letters in columns as indicated by Tukey HSD test at *p* ≤ 0.05. ^ii NS^ indicate that the F values for 50% (10.75 L), 75% (16.13 L), and 100% (21.50 L) are not significant (*p* > 0.05); * indicate that the F values for 50% (10.75 L), 75% (16.13 L), and 100% (21.50 L) are significant at *p* ≤ 0.05. ^iii^ Water consumption before TRT indicates water consumed by plants prior to water volume treatments being applied. Water consumption during TRT refers to the water consumed by plants after different volumes were applied. The total water consumption shows the total volume of water consumed throughout the experiments.

**Table 3:** Root variables analyzed with WinRhizo software 2016a on carrot grown in a deep-water culture system.

Treatment	Total Length	Average Diameter	Root Volume
	(cm)	(mm)	(cm^3^)
Second experiment			
50% (10.75 L) Volume	4136.40	2.77 ^b^	14.46
75% (16.13 L) Volume	3678.38	3.77 ^a^	16.33
100% (21.50 L) Volume	4142.63	2.48 ^b^	15.28
*p*-values	0.501 ^NS^	0.030 *	0.817 ^NS^

^i^ Mean separation is shown by different lowercase letters in columns as indicated by Tukey HSD test at *p* ≤ 0.05. ^ii NS^ indicate that the F values for 50% (10.75 L), 75% (16.13 L), and 100% (21.50 L) are not significant (*p* > 0.05); * indicate that the F values for 50% (10.75 L), 75% (16.13 L), and 100% (21.50 L) are significant at *p* ≤ 0.05.

**Table 4:** Restricted Maximum Likelihood correlation analysis of variables measured at harvest from the deep-water culture carrot with different nutrient solution volumes.

	Aerial Weight (g)	Lateral Root Weight (g)	Tap Root Weight (g)	Max Root Length (cm)	Aerial Height (cm)	Total Water Consumption (L)	Root/Shoot Ratio	LateralRoot/TapRoot Ratio	WUE (g FW/L)	Total Carotenoids (μg/g DW)
Aerial weight (g)	1									
Lateral root weight (g)	**0.8858**	1								
Tap root weight (g)	**0.6716**	**0.5947**	1							
Max Root length (cm)	0.3903	0.4226	**0.5350**	1						
Aerial height (cm)	0.2524	0.2642	0.0855	0.1390	1					
Total water consumption (L)	0.2487	0.3212	0.3041	0.2218	0.2345	1				
Root/shoot ratio	0.2682	**0.4816**	**0.7268**	0.4110	−0.0875	0.3642	1			
LateralRoot/TapRoot ratio	0.4244	**0.6726**	−0.1179	0.0948	0.2381	0.0213	0.0190	1		
WUE (g FW/L)	**0.6628**	**0.5643**	**0.9759**	**0.4899**	0.0159	0.1113	0.6889	−0.129	1	
Total carotenoids (μg/g DW)	**−0.5270**	**−0.5787**	−0.3019	−0.2598	0.0302	−0.1368	−0.2441	−0.4211	−0.2794	1

^i^ Significance levels are formatted as follows: Underline: *p* ≤ 0.05, Bold: *p* ≤ 0.01, Bold and Underline: *p* ≤ 0.001.

**Table 5:** Pairwise correlation analysis of variables measured at harvest from the deep-water culture carrot with different nutrient solution volumes.

	Aerial Weight (g)	Lateral Root Weight (g)	Tap Root Weight (g)	Max Root Length (cm)	Aerial Height (cm)	Total Water Consumption (L)	Root/Shoot Ratio	LateralRoot/TapRoot Ratio	WUE (g FW/L)	Total Carotenoids (μg/g)
Aerial weight (g)	1									
Lateral root weight (g)	**0.9212**	1								
Tap root weight (g)	**0.8517**	**0.8315**	1							
Max Root length (cm)	0.2579	0.2411	0.2755	1						
Aerial height (cm)	**0.6148**	**0.515**	**0.5069**	0.3481	1					
Total water consumption (L)	0.2414	0.1134	0.3125	−0.143	0.2002	1				
Root/shoot ratio	−0.4479	−0.4247	−0.0848	0.0487	−0.3439	0.0611	1			
LateralRoot/TapRoot ratio	**0.4843**	**0.5518**	0.1742	0.3216	0.329	−0.3162	**−0.4668**	1		
WUE (g FW/L)	**0.8397**	**0.8388**	**0.9641**	0.3355	**0.4965**	0.0634	−0.1145	0.2754	1	
Total carotenoids (μg/g)	−0.1346	−0.2938	−0.2868	0.2422	−0.0654	−0.1577	−0.0914	0.0401	−0.2555	1

^i^ Significance levels are formatted as follows: Underline: *p* ≤ 0.05, Bold: *p* ≤ 0.01, Bold and Underline: *p* ≤ 0.001.

## Data Availability

Certain data are available upon request.
